# In-hospital Mortality and the Predictive Ability of the Modified Early Warning Score in Ghana: Single-Center, Retrospective Study

**DOI:** 10.2196/24645

**Published:** 2021-07-12

**Authors:** Enoch Joseph Abbey, Jennifer S R Mammen, Samara E Soghoian, Maureen A F Cadorette, Promise Ariyo

**Affiliations:** 1 Division of Endocrinology, Diabetes and Metabolism Department of Medicine Johns Hopkins School of Medicine Baltimore, MD United States; 2 Department of Emergency Medicine New York University Grossman School of Medicine New York University Langone Health New York, NY United States; 3 Occupational and Environmental Health Department of Environmental Health and Engineering Johns Hopkins School of Public Health Baltimore, MD United States; 4 Division of Adult Critical Care Department of Anesthesiology and Critical Care Medicine Johns Hopkins School of Medicine Baltimore, MD United States

**Keywords:** modified early warning score, MEWS, AVPU scale, Korle-Bu Teaching Hospital, KBTH, Ghana, critical care, vital signs, global health

## Abstract

**Background:**

The modified early warning score (MEWS) is an objective measure of illness severity that promotes early recognition of clinical deterioration in critically ill patients. Its primary use is to facilitate faster intervention or increase the level of care. Despite its adoption in some African countries, MEWS is not standard of care in Ghana. In order to facilitate the use of such a tool, we assessed whether MEWS, or a combination of the more limited data that are routinely collected in current clinical practice, can be used predict to mortality among critically ill inpatients at the Korle-Bu Teaching Hospital in Accra, Ghana.

**Objective:**

The aim of this study was to identify the predictive ability of MEWS for medical inpatients at risk of mortality and its comparability to a measure combining routinely measured physiologic parameters (limited MEWS [LMEWS]).

**Methods:**

We conducted a retrospective study of medical inpatients, aged ≥13 years and admitted to the Korle-Bu Teaching Hospital from January 2017 to March 2019. Routine vital signs at 48 hours post admission were coded to obtain LMEWS values. The level of consciousness was imputed from medical records and combined with LMEWS to obtain the full MEWS value. A predictive model comparing mortality among patients with a significant MEWS value or LMEWS ≥4 versus a nonsignificant MEWS value or LMEWS <4 was designed using multiple logistic regression and internally validated for predictive accuracy, using the receiver operating characteristic (ROC) curve.

**Results:**

A total of 112 patients were included in the study. The adjusted odds of death comparing patients with a significant MEWS to patients with a nonsignificant MEWS was 6.33 (95% CI 1.96-20.48). Similarly, the adjusted odds of death comparing patients with a significant versus nonsignificant LMEWS value was 8.22 (95% CI 2.45-27.56). The ROC curve for each analysis had a C-statistic of 0.83 and 0.84, respectively.

**Conclusions:**

LMEWS is a good predictor of mortality and comparable to MEWS. Adoption of LMEWS can be implemented now using currently available data to identify medical inpatients at risk of death in order to improve care.

## Introduction

Critical illness is a leading cause of morbidity and mortality in sub-Saharan Africa, including Ghana [1]. Low- and middle-income countries have a disproportionately higher burden of critical illness with over 90% of global maternal deaths and deaths from trauma and infections [1-3]. In Ghana, the critical care burden is high. Historically, financial investment has been skewed toward primary health care. Less commitment to critical care means that resources for intensive medical care are limited, and their thought-out and appropriate allocation is important [4].

**Figure 1:**
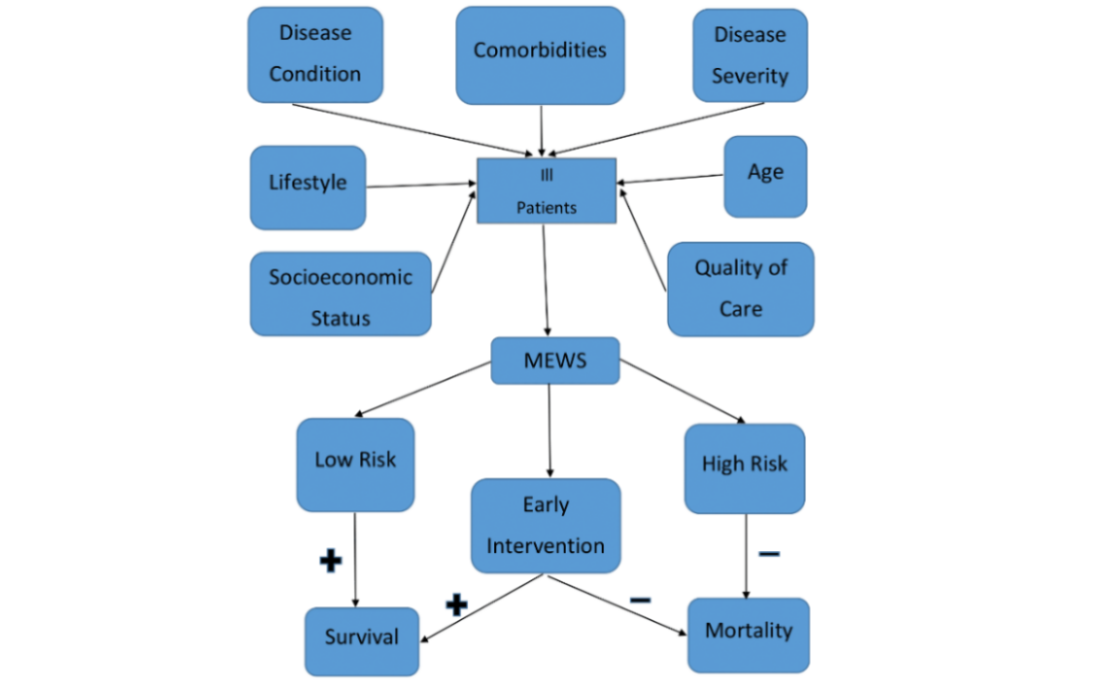
Conceptual framework showing predictors of in-hospital mortality and the role of the modified early warning score (MEWS) among ill patients.

One of the main reasons why patients deteriorate and die in hospitals is delayed recognition of illness severity in understaffed inpatient wards. Early warning tools to help identify patients at the highest risk of death could help countries like Ghana with resource allocation and clinical decision making (Figure 1).

Multiple studies have shown that critical illness and serious adverse events in hospitalized patients are preceded by signs of clinical deterioration in up to 80% of those affected [5-8]. Therefore, changes in physiological parameters can be used to predict adverse events such as shock, cardiac arrest, death, and unplanned intensive care unit (ICU) admissions [9].

MEWS is a commonly used illness severity score that is calculated by combining five physiologic bedside parameters: systolic blood pressure, heart rate, respiratory rate, temperature, and level of consciousness assessed by the AVPU (alert, [responds to] voice, [responds to] pain, unresponsive) scale or RASS (Richmond Agitation Sedation Scale) score. These four vital signs and the observation of consciousness are individually scored and summed to yield a combined score between 0 and 14, with higher scores representing increased illness severity.

In a systematic review conducted by Smith et al [10] in 2014, early warning scores, including MEWS, had strong predictive ability for death and cardiac arrest within 48 hours in academic urban hospitals in economically advanced countries. Early warning scores have also been shown to provide precise, concise, and unambiguous means of identifying and communicating about clinical deterioration to help clinical staff provide special attention and care to patients who need it most (justifiable appropriation of care) [11]. As a result, scoring systems such as MEWS have been adopted in most developed countries and some African countries [12-14].

This study sought to validate the use of MEWS as a clinical decision-making tool to improve early identification of hospitalized medical patients at increased risk for death in Ghana. In addition, since level of consciousness is not routinely recorded in current clinical practice, we aimed to investigate the predictive utility of a limited MEWS (LMEWS) calculation based on vital signs alone. Most studies in similar settings have found that the level of consciousness is generally high (ie, the patient is well oriented) even when other aspects of the MEWS value are abnormal [2]. We therefore hypothesized that the physiologic data currently being monitored in Ghana may be sufficient to improve the early detection of critical illness and help guide resource allocation among inpatients in this setting.

## Methods

### Study Design and Population

**Figure 2:**
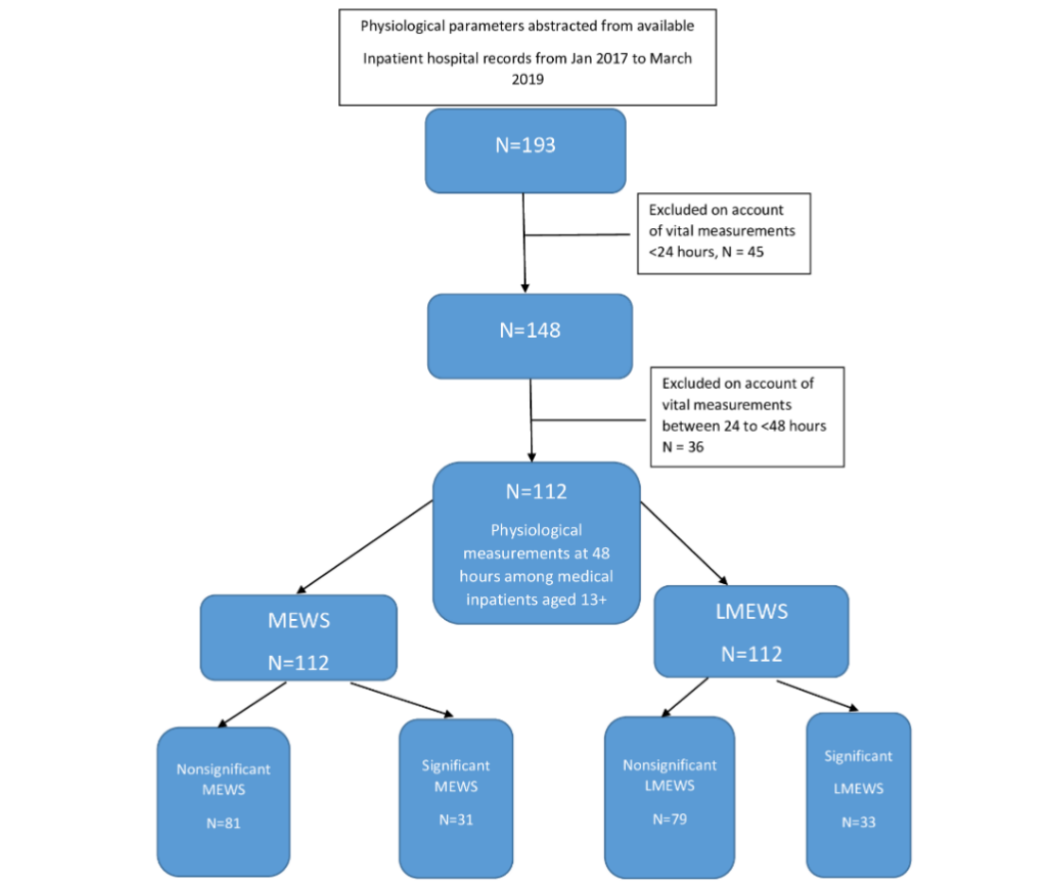
Flow chart demonstrating the creation of the modified early warning score (MEWS) cohort. LMEWS: limited MEWS.

This was a retrospective chart review study of hospitalized medical patients, aged ≥13 years, admitted to the Korle-Bu Teaching Hospital in Accra, Ghana. The Korle-Bu Teaching Hospital is the national hospital of Ghana and the leading tertiary care referral center in the country [15]. Medical inpatients hospitalized there for at least 48 hours whose medical records were still available from the period of January 2017 to March 2019 were included in the study. During this period, the standard practice was to discharge patients in possession of their written medical records; copies were not often retained. This practical limitation accounts for the smaller study size than might be expected for a tertiary facility. Pediatric patients, defined as those aged less than 13 years of age by the Ghana Ministry of Health guidelines, were not included. Patients with more than one hospital admission in the past month, or those who were admitted for conditions other than medical ones, were also excluded (Figure 2). The maximum in-hospital stay was 32 days, and no follow-up data were collected post discharge.

Demographic data were collected to analyze covariates. Patients’ vital signs recorded at 48 hours after admission were recoded and scored to generate the LMEWS value, using thresholds as previously described (Table 1) [2]. To compare the utility of LMEWS with the full MEWS in the absence of routine observation of consciousness and recording of AVPU scores, we generated a full MEWS value using imputation by randomly assigning 92% of the sample to a status of “alert” (AVPU score=0) and the rest to scores between 1 and 3. These percentages were determined based on the findings of a study by Subbe et al [2], which used a similar patient population.

Our study was based on the conceptual framework depicted in Figure 1, which identifies correlational patterns of how different events and experiences may predict mortality in a hospitalized patient. A predictive model was designed using multivariable logistic regression and validated for model accuracy to compare patients with significant MEWS to patients with nonsignificant MEWS, where a significant MEWS was defined as a score ≥4, and a nonsignificant MEWS was defined as a score <4 in the absence of the AVPU [3,16,17]. This cut-off did not vary for the LMEWS versus MEWS values since for most individuals the level of consciousness is normal and therefore contributes 0 points to the total MEWS value.

Due to the confidential nature of patient information, and the need to protect anonymity and obtain consent during health record reviews, ethical approval and waiver of documented permission was obtained from the Institutional Review Board (IRB) of Johns Hopkins University, and from the Scientific and Technical Committee (KBTH-STC 00017/2019) and the IRB of the Korle-Bu Teaching Hospital. Although reporting was anonymous, patients’ records were not, so researchers involved in data collection and handling also signed a confidentiality clause.

**Table 1:** Scoring scale for the modified early warning score (MEWS) adopted form Subbe et al [2].

Physiological parameter	MEWS value						
	3	2	1	0	1	2	3
Systolic blood pressure (mmHg)	<70	71-80	81-100	101-199	—^a^	≥200	—
Heart rate (bpm)	—	41-50	41-50	51-100	101-110	111-129	≥130
Respiratory rate (cpm)	—	—	—	9-14	15-20	21-29	≥30
Temperature (°C)	—	—	—	35-38.4	—	≥38.5	—
AVPU^b^ score	—	—	—	Alert	Reacting to voice	Reacting to pain	Unresponsive

^a^Not applicable.

^b^AVPU: alert, voice, pain, unresponsive.

### Statistical Analysis

Data were analyzed using STATA (version 15.1, StataCorp LLC). The estimated sample size was determined a priori based on work by Kyriacos et al [18], which yielded a minimum sample size of 46 based on a significance level of .05, delta value of 0.45, and power of 80% to detect clinical deterioration in postoperative patients using MEWS. Post–data collection power analysis was also performed, based on a chi-square test comparing two independent proportions. Based on the resulting analytic sample of 112 participants, with 31 in the significant MEWS category and 81 in the nonsignificant MEWS category, our study achieves a power of 95% to detect a difference in outcome percentages of at least 37% between these two groups. Testing for associations with survival to discharge versus in-hospital mortality was conducted using a two-sample *t* test for each of the individual continuous physiological parameters. The chi-square test was used to test for differences in the proportion of patients with each outcome in the categories of significant versus nonsignificant MEWS and LMEWS. Univariable log-binomial regression analysis was used to estimate unadjusted risk ratios between each predictor and mortality. Multivariable Poisson regression with robust variance was used due to the failure of convergence of the log-binomial regression model. Logistic regression analysis (odds ratio [OR]) was used to identify an appropriate predictive model. A *P* value of <.05 was considered statistically significant. The accuracy of the prediction model was determined using the receiver operating characteristic (ROC) curve and C-statistic (where a C-statistic of 0.5 implies the model performs no better than random chance and a score of 1.00 perfectly discriminates between categories). Adjustment was made for the following potential confounders: age, sex, duration of admission, admission to the ICU, presence or absence of other comorbidities, and the organ system involved in the disease process. The Hosmer-Lemeshow test was used to determine model fit for both the MEWS and LMEWS models, with *P* values ≥.05 implying satisfactory fit. A sensitivity analysis was done using a cut-off of ≥5 to distinguish significant from nonsignificant MEWS and LMEWS values. Missing values were limited to the reason for admission (organ system) and represented <1% (1/112).

## Results

The sample comprised 112 patients admitted for medical reasons during the study period. Of these, 62% (69/112) were male with a mean age of 47 years (SD 17.5), and 38% (43/112) were female with a mean age of 52 years (SD 20) (Table 1). Overall mortality was 41.1% (46/112) and increased with age. Every year increase in age was associated with a 3% increase in mortality rate after adjusting for MEWS (IRR [incidence rate ratio]=1.03, 95% CI 1.02-1.04). For patients who survived, the most common admission diagnoses were genitourinary system abnormalities (17/65, 26.2%), whereas neurologic conditions were most common among patients who died (18/46, 39%). The longest length of in-hospital stay was 32 days, with an average of 8 days.

At 48 hours post admission, patients’ mean systolic blood pressure was 125 mmHg (SD 2.9), average pulse rate was 91 mmHg (SD 2), mean axillary temperature was 36.9°C (SD 0.1), and average respiratory rate was 24 cpm (SD 4.7). Only temperature and respiratory rate were individually associated with mortality (Table 2). Physiological parameters measured at 48 hours produced an average LMEWS value of 3 (range 0-11). Imputation of randomly assigned AVPU values increased mean scores by 8% overall, producing an average MEWS of 3 (range 0-14).

A significant MEWS was associated with a relative risk of 2.01 (95% CI 1.33-3.04) for death in the univariable analysis, while a significant LMEWS had a relative risk of 2.19 (95% CI 1.46-3.30) in the univariable analysis (Table 3).

**Table 2:** Showing baseline characteristics.

Characteristic		Survival to discharge (n=66)	Death in hospital (n=46)	*P* value^a^	
Sex (male), n (%)		45 (68.2)	24 (52.2)	.09	
**Age (years), n (%)**					<.001
	25-64	46 (69.7)	27 (58.7)		
	≥65	7 (10.6)	18 (39.1)		
**Disease type by system involved, n (%)**					.01
	Cardiopulmonary	15 (23.1)	13 (28.3)		
	Neuroendocrine	11 (16.9)	18 (39.1)		
	Hemaoncological	11 (16.9)	1 (2.2)		
**Physiological parameter at 48 hours, mean (SD)**					
	Systolic blood pressure (mmHg)	127.8 (29.4)	120.7 (32.1)	.23	
	Pulse rate (bpm)	89 (17.6)	94 (18.1)	.17	
	Axillary temperature (°C)	36.7 (0.7)	37.3 (1.2)	.002	
	Respiratory rate (cpm)	23 (4.7)	25 (6.9)	.03	
	Average length of admission	7 (6.3)	8 (7)	.60	

^a^*P* values obtained via the *t* test and the chi square test.

**Table 3:** Multivariable logistic regression of death using full modified early warning score (MEWS) and the limited MEWS (LMEWS).

Covariate	MEWS, odds ratio (95% CI)	LMEWS, odds ratio (95% CI)
Age	1.08 (1.04-1.12)	1.08 (1.04-1.12)
Sex (male)	0.44 (0.16-1.23)	0.40 (0.14-1.13)
MEWS (significant)	6.33 (1.96-20.49)	8.22 (2.45-27.56)
Duration of admission	0.99 (0.93-1.07)	1.01 (0.94-1.08)
Diseased organ system	0.59 (0.31-1.13)	0.59 (0.31-1.12)

The death rate calculated by the Poisson regression after adjusting for only age was 2.02 (95% CI 1.40-2.91) times higher in patients with a significant MEWS compared to those with a nonsignificant MEWS. The death rate for a significant MEWS value using LMEWS was 2.13 (95% CI 1.48-3.07) times that of nonsignificant MEWS after adjusting for age.

In the multivariable predictive model adjusting for age, sex, duration of admission, admission to the ICU, organ system involved, and comorbidities, the odds of death among patients with a significant MEWS was 6.33 (95% CI 1.96-20.50) times that of patients with a nonsignificant MEWS. The death rate among patients with a significant LMEWS was 8.2 (95% CI 2.5-27.6) times that of patients with a nonsignificant LMEWS in the multivariable analysis. The best multivariable regression model was selected based on the Akaike Information Criteria, with a value of 116.4. The odds of death for every year increase in age was 8% (OR 1.08, 95% CI 1.04-1.12). Other covariates were not statistically significant.

**Figure 3:**
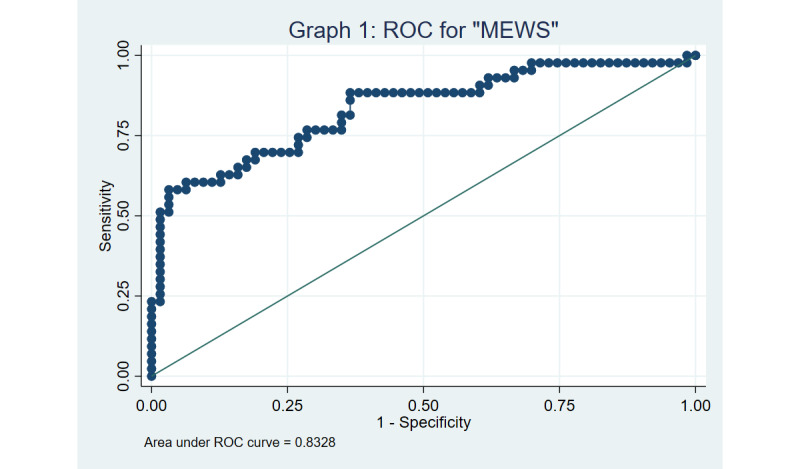
Receiver operator characteristic (ROC) curve for the modified early warning score (MEWS) using a cut-off of 4.

**Figure 4:**
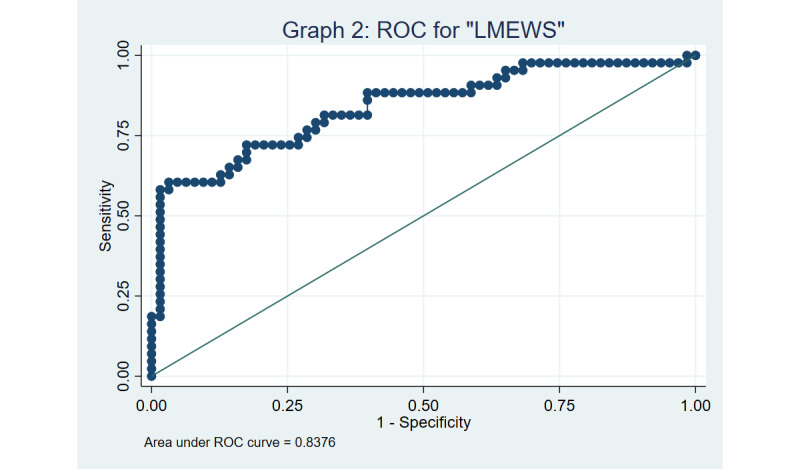
Receiver operator characteristic (ROC) curve for the limited modified early warning score (LMEWS) using a cut-off of 4.

Both MEWS and LMEWS were found to have good discrimination based on the ROC curves, with a C-statistic of 0.833 and 0.838, respectively (Figures 3 and 4), using a cut-off of ≥4. The Hosmer-Lemeshow goodness-of-fit test yielded *P* values of .16 and .25 for MEWS and LMEWS, respectively, implying that our model fits the data well (the null hypothesis being that the prediction model is correctly specified).

**Figure 5:**
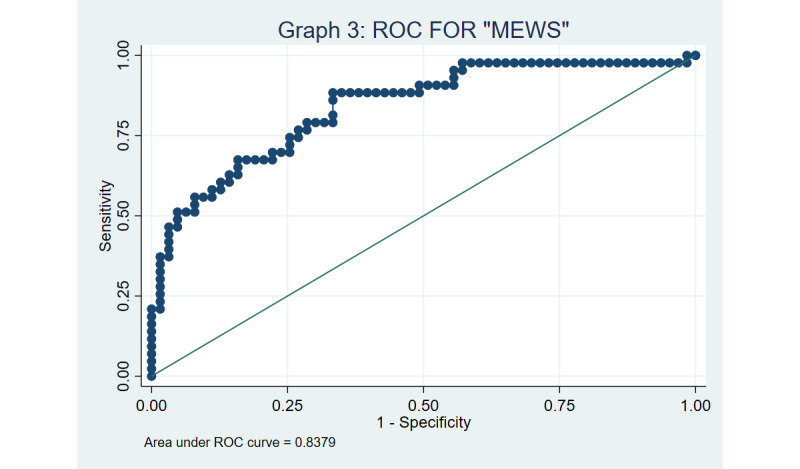
Receiver operator characteristic (ROC) curve for the modified early warning score (MEWS) using a cut-off of 5.

**Figure 6:**
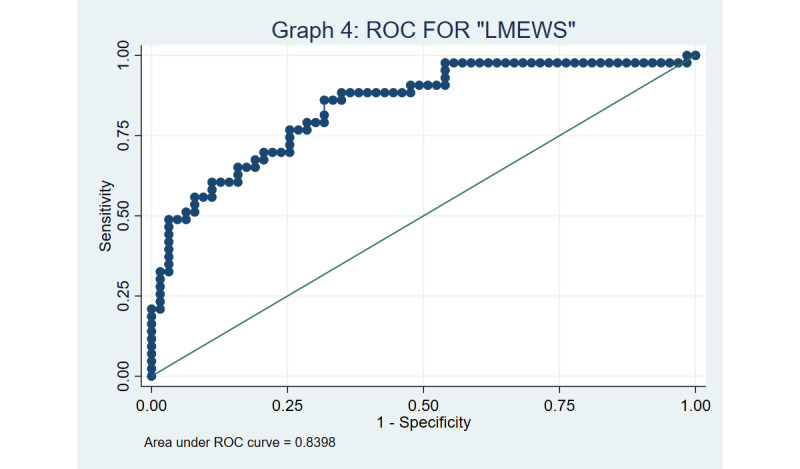
Receiver operator characteristic (ROC) curve for the limited modified early warning score (LMEWS) using a cut-off of 5.

Sensitivity analyses using a significant MEWS or LMEWS cut-off score of ≥5 yielded a multivariable OR of 12.4 (95% CI 2.5-61.2) and 15.1 (95% CI 2.5-91.8), respectively. The ROC curves for MEWS and LMEWS was found to be 0.838 and 0.840, respectively, when a cut-off of ≥5 was adopted, as captured in Figures 5 and 6. The Hosmer-Lemeshow test to assess goodness of fit yielded *P* values of .51 versus .77 for MEWS and LMEWS, respectively, when a cut-off of ≥5 was used.

## Discussion

### Principal Findings

MEWS has been validated in several settings as a robust predictor of both clinical deterioration and death in hospital [2,18]. This study demonstrates that the approach is useful even in the absence of an observed level of consciousness. Vital signs data collected routinely at the bedside in most facilities in Ghana and throughout sub-Saharan Africa can be used to generate LMEWS, which also has a high predictive value.

Serious adverse events and some portion of in-hospital mortality can be prevented by limiting human error, such as failure to recognize the early warning signs of a deteriorating patient or failure to act on this information in a timely manner [19]. MEWS is a low-cost tool that utilizes easy-to-measure bedside parameters to generate a singular value that can identify at-risk patients. This value can be used as a preset trigger in the context of a reporting algorithm.

We found that, in this setting, having a LMEWS value of 4 or greater was highly associated with in-hospital mortality. The area under the curve (AUC) of 0.84 for the LMEWS is consistent with good model accuracy in the discrimination of patients who are critically ill. The combination of LMEWS with clinical judgment is therefore likely to be as effective in Ghana as it has been in other similarly resourced settings [20]. This is encouraging since LMEWS can be implemented without additional training of staff on how to score the level of consciousness and without changing standardized documentation forms already in use for patient monitoring.

The standard inpatient vital signs monitoring charts used in many Ghanaian hospitals includes a 4-hourly graphic to plot temperature, pulse rate, respiratory rate, and blood pressure. Additional parameters may also be serially recorded in some instances or centers; however, the typical bedside observation chart does not record the level of consciousness for patients, as captured in the MEWS by including either the AVPU or RASS score.

Although the original description defined a significant MEWS as any single score ≥5, or any increase of 2+ points in patients with initial scores above 5, a cut-off of 4 was adopted for this study [2,16]. Arguably, a lower threshold for detection would increase the burden of patient re-examination and reassessment on health care providers, potentially making use of the score impractical in settings with severely limited human resources. The decision to adopt a cut-off score of 4 as the definition of a significant MEWS was based on previous work done by Gardner-Thorpe et al [16] in 2006, which showed that raising the threshold reduces the sensitivity to unacceptable levels for patient safety, though an increase in specificity would be observed. Using a cut-off of 4, the number of individuals with a significant MEWS value was 33 (out of 112), and 31 had a significant LMEWS value. In other words, nearly 30% of the patients in our study would have been categorized as high risk for clinical deterioration in the context of a MEWS-based reporting algorithm.

Interestingly, using MEWS or LMEWS with a cut-off of ≥5 did not only yield higher discrimination, based on the C-statistics, but also had better calibration in terms of correctly assessing the risk of disease severity. Based on the receiver operating characteristics and the Hosmer-Lemeshow goodness-of-fit test, LMEWS with a cut-off of ≥5 was superior to both MEWS and LMEWS with a cut-off of ≥4.

Encouraging complete, accurate documentation and a standardized interpretation of vital signs with appropriate actions by nurses, doctors, and other allied staff can potentially improve the outcomes of patients admitted to hospitals, even in a setting that lacks rapid response teams. Many interventions such as fluids or antibiotics do not require advanced equipment or costly supplies, making the implementation of the afferent arm of a rapid response system important even in settings where the efferent arm is more limited [21].

### Limitations

This study is subject to all the limitations of a single-center, retrospective chart review. Sources of bias include the potential for differential clinical care based on perceived patient status in the absence of a standardized rapid response team or protocol. In addition, the study only examined vital signs collected at a single time point for each patient. Changes in serially measured physiological parameters were not evaluated. A study published by Ludikhuize et al [22] recommends the calculation of MEWS at least 3 times daily to detect the development of physiological abnormalities. Our study could not have detected any significant MEWS values that may have developed after the first 48 hours upon admission. However, missing additional patients who may have worsened later and then died would bias the study toward the null hypothesis. This makes our study design a conservative one, with results consistent with previously published literature on the topic [2,16].

More prospective research is needed to help define the utility of LMEWS for physicians looking to allocate resources and develop rapid response teams that can act on predictive information to improve patient outcomes and patient care.

### Conclusion

This study was the first to examine the ability of an early warning system to predict inpatient mortality based on routinely collected clinical data in a low-resource setting. Early recognition of clinical status decline is critical even in low-resource settings, where bedside interventions may prevent ICU admissions and disease complications including death. Though the MEWS system provides good discrimination, the LMEWS provides better discrimination and calibration in the prediction of mortality and can identify critical illness among inpatients with primarily medical diagnoses. Additional prospective studies will be useful to validate LMEWS among other categories of inpatients and to investigate its impact on health resource allocation and clinical outcomes in low-resource settings.

## Abbreviations

AUC: area under the curve
AVPU: alert, voice, pain, unresponsive
ICU: intensive care unit
IRB: Institutional Review Board
IRR: incidence rate ratio
LMEWS: limited modified early warning score
MEWS: modified early warning score
OR: odds ratio
RASS: Richmond Agitation Sedation Scale
ROC: receiver operating characteristic
